# Self-Renewal and Differentiation Capacity of Urine-Derived Stem Cells after Urine Preservation for 24 Hours

**DOI:** 10.1371/journal.pone.0053980

**Published:** 2013-01-18

**Authors:** Ren Lang, Guihua Liu, Yingai Shi, Shantaram Bharadwaj, Xiaoyan Leng, Xiaobo Zhou, Hong Liu, Anthony Atala, Yuanyuan Zhang

**Affiliations:** 1 Department of Hepatobiliary Surgery, Beijing Chaoyang Hospital, Capital Medical University, Beijing, People's Republic of China; 2 Wake Forest Institute for Regenerative Medicine, Wake Forest School of Medicine, Winston-Salem, North Carolina, United States of America; 3 Department of Biostatistical Sciences, Wake Forest School of Medicine, Winston-Salem, North Carolina, United States of America; 4 Radiology/Translational Biology Department, The Methodist Hospital Research Institute, Houston, Texas, United States of America; 5 Center for Bioengineering and School of Electrical and Computer Engineering, University of Oklahoma, Norman, Oklahoma, United States of America; Instituto Butantan, Brazil

## Abstract

Despite successful approaches to preserve organs, tissues, and isolated cells, the maintenance of stem cell viability and function in body fluids during storage for cell distribution and transportation remains unexplored. The aim of this study was to characterize urine-derived stem cells (USCs) after optimal preservation of urine specimens for up to 24 hours. A total of 415 urine specimens were collected from 12 healthy men (age range 20–54 years old). About 6×10^4^ cells shed off from the urinary tract system in 24 hours. At least 100 USC clones were obtained from the stored urine specimens after 24 hours and maintained similar biological features to fresh USCs. The stored USCs had a “rice grain” shape in primary culture, and expressed mesenchymal stem cell surface markers, high telomerase activity, and normal karyotypes. Importantly, the preserved cells retained bipotent differentiation capacity. Differentiated USCs expressed myogenic specific proteins and contractile function when exposed to myogenic differentiation medium, and they expressed urothelial cell-specific markers and barrier function when exposed to urothelial differentiation medium. These data demonstrated that up to 75% of fresh USCs can be safely persevered in urine for 24 hours and that these cells stored in urine retain their original stem cell properties, indicating that preserved USCs could be available for potential use in cell-based therapy or clinical diagnosis.

## Introduction

Although preservation of various organs and tissues in protective solutions at low temperature (4°C) for 24 hours and cryopreservation of cells in liquid nitrogen have been successfully established [1], [2], the storage of stem cells in body fluid has not yet been explored. We have recently found stem cells in urine (termed urine-derived stem cells, or USCs) that possess a high capability for expansion and multi-potent differentiation properties toward osteocyte, chrondocyte, adipocyte, myocyte, endothelial and urothelial cells [3], [4], [5], [6], [7]. To develop a reliable method of preservation of body fluid-derived stem cells, such as USCs, preservation of stem cells in urine would enable a maximum amount of high-quality donor cells in a short period of time and alleviate damage by storing the cells in urine. USCs at earlier passages have more potential for self-renewal and differentiation; thus, it would be an advantage to generate more of these cells at early passages (such as p2 or p3) within a short period of time (7–10 days). Patients' urine samples could be transferred from home to the hospital. This method would also improve cell transportation or distribution from sites where cell isolation is not immediately available, to places where cell isolation and cell culture can be done. USCs can be obtained via a simple, safe, non-invasive, reliable and low-cost approach, and their use has great potential for clinical application. USCs might be a viable cell source for cell therapy and tissue engineering in urology, such as cell therapy for the treatment of stress urinary incontinence [7], [8], vesicoureteral reflux, or bladder and urethra tissue engineering [4], [9], and in other fields as well.

The purpose of this study was to determine whether USCs still possess stem cell features and functions after being stored in urine at 4°C for 24 hours. We determined the total number of cells shed off from entire urinary tract system into the urine within 24 hours; we also optimized preservation methods to retain the maximum number of high-quality USCs. We then characterized the preserved stem cells 24 hours after urine storage, and compared them to fresh USCs with regard to cell morphology, cell growth patterns, population doubling, stem cell surface marker expression, telomerase activity, karyotypes, myogenic protein marker expression, contractility of myogenic differentiated USCs, urothelial protein marker expression, tightness of junctions, and barrier function of urothelial differentiated USCs.

## Materials and Methods

### Ethics statements

This study was approved by the Wake Forest University Institutional Review Board (IRB00014033). Written informed consents have been obtained and were approved by Wake Forest University institutional review board.

### Collection of Urine Samples

A total of 415 urine samples were collected from 12 healthy adult men (age range 20–54 years old). Two types of cells were investigated in this study: i.e. urine derived cells (total numbers of cells in the urine) and USCs. To determine total numbers of cells shed into the urine (urine derived cells) in 24 hours, 166 urine specimens were used on 3 consecutive days (24 h×3 d). The cells were stained with trypan blue and counted. A total of 189 urine samples were preserved in seven different preservation solutions at 4°C for 24 hours. Group 8 specimens (total of 9) were only preserved for 12 hours (Table 1). To evaluate the effect of preservation solution on cell survival and function of USCs, several solutions were tested: 1) histidine-tryptophan-ketoglutarate (HTK) solution; 2) University of Wisconsin (UW) solution; 3) culture media (a mixture of keratinocyte-serum free medium [KSFM] and embryonic fibroblast medium [EFM]) with a final concentration of 0.5% fetal bovine serum (FBS) for USCs [3]; 4) culture media with 10% FBS; 5) culture media without FBS; 6) 0.5% FBS alone and 7) culture media with 0.5% FBS for a 12 hour. All preservation solutions were used at 10% (v/v), e.g. 25 ml solution in 225 ml voided urine. Urine samples were also stored without any preservation solution as a negative control. Cells from fresh urine samples were cultured as a positive control. All donors had no urinary tract infection.

**Table 1:** Preservation solutions and methods to store the urine samples for 24 hours period.

Preservation solutions	Storage Procedures
**G1**: Fresh urine sample (as control)	10% USC culture media in urine sample with no storage. Cells were harvested and cultured immediately.
**G2.** HTK solution	10% HTK solution in voided urine sample at 4°C for 24 hours (25 ml HTK in 225 ml urine).
**G3**: UW solution	10% UW solution in urine sample at 4°C for 24 hours.
**G4**: USC culture medium (0.5% serum)	10% USC with 5% FBS culture media in urine sample (0.5% serum) at 4°C for 24 hours.
**G5**: USC culture media (10% FBS)	10% USC culture media and add extra 9.5% FBS in urine sample (final FBS concentration is 10%) at 4°C for 24 hours.
**G6**: USC culture medium (serum free)	10% USC culture media in urine sample (no serum) at 4°C for 24 hours.
**G7**: 0.5% FBS	0.5% serum in urine sample at 4°C for 24 hours.
**G8.** USC culture medium (0.5% FBS)	10% USC with 5% FBS culture media in urine sample (0.5% serum) at 4°C for 12 hours.
**G9**: No preservation solution (as control)	No preservation solution was used with the urine sample at 4°C for 24 hours.

### Specific Gravity of Urine Samples and Preservation Solutions

Nine urine specimens from three different donors and the five different preservation solutions were used to detect specific gravity at room temperature and after storage at 4°C for 24 hours. Specific gravity was measured by a Mettler Toledo Densito 30PX device (Schwerzenbach, Switzerland) following the manufacturer's instructions.

### Culture of Urine-Derived Cells

For isolation of cells from fresh urine samples and preserved urine samples after storage at 4°C for 24 hrs, the urine samples were centrifuged at 500×g for 5 min at room temperature. The cell pellets were re-suspended and then plated into 24-well tissue culture plates (Becton Dickinson, Franklin Lakes, NJ). The medium used was composed of keratinocyte-serum free medium (KSFM, Invitrogen, Carlsbad, CA) and embryonic fibroblast medium (EFM) at a 1∶1 ratio with 5% FBS [3]. Urine-derived cells were isolated and characterized as previously described [3], [5]. Numbers of cell clones were counted from each urine sample after they were cultured for 3 weeks. Cell colonies from fresh urine samples often appeared as a cluster of 5–12 cells within 5–7 days after plating on the cell culture wells. However, cell colonies from 24-hr preserved urine samples appeared 7–8 days after being initially plated. The cells were allowed to grow to a confluence of 50–70% before subculture. A single cell clone per well was used for further experimentation; further colonies in one well were discarded. Cell morphology at each passage, population doublings (PD), cell growth pattern, expression of mesenchymal stem cell surface markers, relative telomerase activity, and bi-potential differentiation capability from fresh and 24-hr preserved urine samples were assayed when USCs were at passage 2.

### Cell Proliferation

USCs from fresh and 24 hr preserved culture urine samples (P2) were seeded in 24-well plates at a density of 1,250 cells/cm^2^. The culture medium was changed every other day. Cell proliferation was measured on days 1, 3, 5 and 7 using an MTS assay (Promega). Briefly, the MTS reagent was incubated with the cells in the dark for 1 hour at 37°C. Following the incubation, 100 µl of the supernatant was transferred to a 96 well micro-plate and the absorbance was measured at 490 nm using a spectrophotometer (Molecular Devices Inc, Sunnyvale, CA). Repeated measurements (n = 5) were carried out for each time point. To calculate PD and doubling time (DT), cell numbers and culture time were counted at each passage from p0 onwards in fresh and 24 hr preserved samples. PD and DT were calculated using the following formula:

PD = ln(N_f_/N_i_)/ln(2); DT = C_t_/PD (N_f_: Final number of cells, N_i_: Initial number of cells, C_t_: Culture time)

### Flow Cytometry

Fresh USCs and 24-hr cultured USCs undergoing exponential growth at passage 2 were stained with specific anti-human antibodies labeled for CD45-FITC, CD31-FITC, CD73-PE, CD90-FITC, CD105-PerCP-Cy™5.5, CD34-FITC, CD44-FITC and CD146-PE. Briefly, cells were trypsinized and 5.0×10^5^ cells were washed and re-suspended in ice-cold PBS containing 1% bovine serum albumin (BSA). Fluorochrome-conjugated antibodies (Table S1) were added to cells in 50 µl PBS containing 3% BSA and incubated on ice for 30 min in the dark. IgG1-PE, IgG1-FITC, IgG2b-FITC and IgG1- PerCP-Cy™5.5 conjugated isotype control antibodies were used to determine background fluorescence. Cells were then washed twice in wash buffer, passed through a 70 µm filter, and analyzed by flow cytometry (FACS Calibur BD Biosciences, Franklin Lakes, NJ).

### Telomerase activity

A total of 30 USC clones collected from fresh and 24-hr preserved urine samples (preserved in USC media containing 0.5% serum and 10% serum) were used for this study. Each group consisted of 10 samples. Whole cell lysates from 2×10^5^ USCs (passage 2) were assayed for telomerase activity using the Telo TAAGG ELISA kit (Roche Applied Sciences, Upper Bavaria, Germany), according to the manufacturer's instructions. Telomeric repeats (TS) 8 provided in the kit was used as a positive control. Samples were considered to be positive for telomerase activity when the difference in absorbance was at least twice that of the negative control.

### Karyotype

Both USC clones from fresh urine samples and 24-hr preserved samples were analyzed for chromosomal stability at passage 4. Briefly, the cultured cells were treated with a hypotonic solution and fixed using methanolacetic acid solution. The metaphase spread on glass slides were digested using trypsin and followed by Giemsa staining to generate G bands along each chromosome. Standard cytogenetic analysis was carried out on the captured images and karyotyping performed using CytoVision software (Leica, Buffalo Grove, IL).

### Urothelial and Smooth Muscle Differentiation of USCs

To confirm that one single USC clone gave rise to two bladder cell lineages, smooth muscle cells (SMC, mesoderm) and urothelial cells (UCs, endoderm), fresh USCs and 24-hr preserved USCs were used from initially-generated single clones. USCs (p2) were plated at a density of 1000 cells/cm^2^ and then induced to differentiation into SMCs and UCs by culture in specific induction media for 14 days. For myogenic differentiation, equal volumes of DMEM and EFM containing 2.5 ng/ml transforming growth factor (TGF-β1) and 5 ng/ml of platelet-derived growth factor (PDGF-BB) was used [5]. For urothelial differentiation, DMEM and KSFM mixed at a 4∶1 ratio containing 30 ng/ml epidermal growth factor (EGF) were used [5]. Final serum concentrations were maintained at 10% and 8% in the myogenic and urothelial differentiation medium, respectively. The differentiation medium was replaced every third day. All growth factors were purchased from R&D Systems (Minneapolis, MN). Cell morphology was recorded before and after growth factor addition for up to 14 days.

### Immunofluorescence

The fresh USCs and 24 hr preserved USCs were induced to differentiate into UCs or SMCs on 8-well chamber slides (Thermo Scientific, Waltham, MA) for 14 days and then immunofluorescent staining with uroepithelial cell markers (Uroplakin Ia and III, cytokeratin (CK-7, CK-13 and CK-20) or smooth muscle cell markers (desmin, myosin, α-SM actin, smoothelin, and calponin) was done (Table S1). The slides were fixed with freshly prepared 4% paraformaldehyde for 15–20 min at room temperature. After removing excess fixative with PBS washes, the cells were permeabilized with 0.1% Triton-X100 in PBS for 10 min and blocked with serum-free block solution (Dako, Denmark) for 15 min. Lineage-specific primary antibodies were diluted appropriately in the antibody diluent solution (Dako) and incubated overnight at 4°C. Unbound primary antibody was removed by using three times PBS washes. Appropriate secondary antibody conjugated to fluorescein-isothiocyanate (FITC, Vector Laboratories, Burlingame, CA) were used to visualize hybridization (green) to primary antibody and incubated at room temperature for 30–45 min in the dark. The slides were mounted using antifade mounting media (Vector Laboratories) containing propidium iodide (PI), and images were captured using a Leica upright microscope (DM 4000B, Germany).

### Western Blot

Various cultured cells (non-induced USCs, urothelially differentiated USCs, myogenically differentiated USCs, normal human ureter urothelial or smooth muscle cells as control) were harvested from each 10 cm culture dish, respectively, and the proteins were extracted using RIPA lysis buffer (Thermo) containing proteinase inhibitor cocktail (Complete mini; Roche Applied Sciences). Protein extracts (15–20 µg) were run on 8–10% sodium dodecyl sulfate-polyacrylamide gels to separate the proteins. A pre-stained protein ladder (Benchmark, Invitrogen) was used to monitor resolution of protein bands. After electrophoresis, the separated proteins were transferred to a PVDF membrane (Millipore, Billerica, MA) by semi-dry transfer. Following transfer, the membrane was incubated in blocking solution (5% nonfat dry milk in PBS) for 1 h at room temperature. For protein signal analysis, the membrane was incubated overnight at 4°C with primary antibodies (Table S1) in 1% milk in PBS, subsequently washed with 0.05% Tween and then incubated with the appropriate diluted HRP-conjugated secondary antibody for 1 hour at room temperature. The blots were visualized with an enhanced chemiluminescence detection system by an enhanced chemiluminescence assay (Supersignal West Femto, Thermo Scientific, Rockford, IL).

### Cell-collagen lattice contractility

To measure cell contractile function of smooth muscle differential USCs *in vitro*, non-induced USCs, myogenically differentiated USCs, and normal human SMCs (control) (3×10^5^cells/mL) were mixed with soluble collagen I (1 mg/mL) neutralized by NaOH, F-12 and NaHCO_3_ (Sigma) to create a USC-collagen I solution. An aliquot (250 µL) of the cell-collagen I solution was placed onto a 35-mm tissue culture plate [5], [10], allowed to set and maintained for 7 days with or without myogenic growth factors. The cell-collagen lattice was then mechanically released from the underlying plastic. The diameters of the cell-collagen lattices were measured before and after releasing (10 min), and the relative change in diameter calculated. Each experiment was performed in triplicate. SMCs-lattices released in the presence of serum (10% FBS) and agonist (10 uM calcium-ionophore, Sigma), served as positive controls; lattices released under serum-free conditions served as negative controls. For the negative controls, cell-collagen lattices were washed twice with serum-free medium to remove any residual serum and incubated for an additional 5 minutes before release. The percentage of contraction was calculated as follows: (Du-Dr)/Du X100 (Du and Dr are the diameters of unreleased lattice and released lattice, respectively).

### Barrier function of urothelially differentiated USC

To assess the barrier function of urothelially differentiated USCs, cells were cultured on 0.4 µm trans-well inserts (Becton Dickinson) and placed in 6-well dishes as reported earlier [11] with minor modifications. Briefly, the inserts were coated with collagen-IV (3 µg/cm^2^), air dried in a laminar hood and sterilized by a 70% ethanol rinse. The ethanol was allowed to evaporate completely before the inserts were used. After USCs (1×10^5^/cm^2^) were plated in the 6-well plate inserts (top chamber) and maintained for 7 days with or without epidermal growth factors (EGF). The 0.5 ml of tracer-containing medium (1 mg/ml, FITC-dextran-FD4, Sigma) was changed in the insert (top chamber) and 3 ml tracer-free medium was added in the bottom well. Phenol-free medium was used to avoid interference of the indicator in the assay. A day before the tracer was added, the media was supplemented with 2 mM CaCl_2_ solution to promote multilayer formation by the differentiated USCs. Media aliquots (150 µl) were removed from the bottom chamber for fluorescence measurements (excitation at 490 nm and emission at 520 nm) after 3 hours. Diffusion of these dyes through a nude insert membrane served as an internal control. Non-induced USCs and normal human urothelial cells were used as negative and positive controls, respectively.

### Transmission electron microscopy (TEM)

TEM studies were used to visualize the presence of tight junctions after induction of USCs to urothelial-like cells. The cells were cultured and induced as described above on 6-well plates trans-well inserts, fixed, and sectioned according to standard TEM protocols [12]. Briefly, the cultured cells were fixed in 2.5% glutaraldehyde, postfixed with 1% osmium tetroxide, dehydrated in graded alcohols, embedded in Spurr's resin (Polysciences, Warrington, PA), and cut into 80-nm sections with a Reichert Ultracut E ultramicrotome. The specimens were viewed and photographed with a Tecnai Spirit BioTwin transmission electron microscope (FEI, Hillsboro, OR) equipped with an AMT 12 megapixel 2 Vu camera (Woburn, MA).

### Statistical analyses

Values are expressed as mean ± standard deviation (SD). One-way ANOVA followed by a Student-Newman-Keuls post hoc test for multiple comparisons were used when appropriate. Pearson correlation analyses were used to study the relationship between total/living cell numbers and age, height. SPSS16.0 software was used for analyses. P≤0.05 was considered as statistically significant.

## Results

### The total number of cells and ratio of living cells after 24-hour preservation in urine

No bacterial contamination was found in any urine samples. The average total number of cells derived from 166 urine samples from 12 healthy adult donors was 58,560±27,980 cells (range 25,226 to 107,543 cells) in 1,680±846 ml urine in a 24-hr period. The average total number of living cells in that period was 8,076±4,784 (range 3,070 to 15,820 cells), as measured by trypan blue exclusion. The ratio of live cells to total cells was 13.65±4.96% (range 5.96%∼22.53%) (Table S2). Pearson correlation analyses showed that number of living cells in the urine appeared to be correlated with age (r = 0.58, *p* = 0.06). Live cell/total cell ratio also appeared to be correlated with height (r = 0.56, *p* = 0.07). The boundary statistically significant *p* values might be due to the small sample size. No other trend of association was found.

### Preserved USCs at 4°C

The preserved USCs initially appeared as a single cell with a rice grain-like appearance after the first 3–5 days. They then formed cell clones with a cluster of 7–8 cell clones, compared to fresh USCs at 2–3 days for single cells and 5–7 days for the clones. The average number of USC clones in every 100 ml urine sample is shown in Table 2. No clones were formed when urine was preserved in UW preservation solution and preservation-free urine sample groups. Among all preservation methods, the USC culture medium with 0.5% serum or 10% serum had the best results (Table 2). USC clones were able to be obtained from each individual. The gross success rate for generating USCs was highest (83%) from fresh samples, and the success rate from urine specimens stored for 24 hr was 70–73%. Although there were differentiating cells in each of above groups, the differentiating cells stopped proliferation, died and were washed away from culture dishes after 1–2 passages.

**Table 2:** Numbers of USC clones in different preservation solutions of urine.

Groups	Gross success rate of generating USC clone (%)	Numbers of cell clones/100 ml urine		Total clones/100 ml
		USC clones	Differentiating cells	
**G1**. Fresh urine (control)(24 hours storage, n = 30)	(83%) 25/30 urine samples	6.29±4.6	1.39±1.47	7.62±5.86
**G2**. HTK solution(24 hours storage, n = 30)		2.07±1.42	0.85±0.54	2.68±1.93
**G3**. UW solution(24 hours storage, n = 30)		0	0	0
**G4**. USC culture medium (0.5% FBS) (24 hours storage, n = 30)	(70%) 21/30 urine samples	3.16±1.95	0.98±0.72	3.91±2.50
**G5**. USC culture medium (10% FBS) (24 hours storage, n = 30)	(73%) 22/30 urine samples	3.37±1.29	0.73±0.32	4.04±1.55
**G6**. USC culture medium (serum free) (24 hours storage, n = 30)		1.08±0.5	1.01±0.48	2.09±0.75
**G7**. 0.5% FBS(24 hours storage, n = 30)		1.19±0.36	0.69±0.29	1.86±0.57
**G8**. USC culture medium (0.5% FBS) (12 hours storage, n = 9)		4.15±1.21	1.29±0.42	5.44±1.39
**G9**. No preservation (control)		0	0	0

### Specific gravity of urine samples and preservation solutions

**Figure 1:**
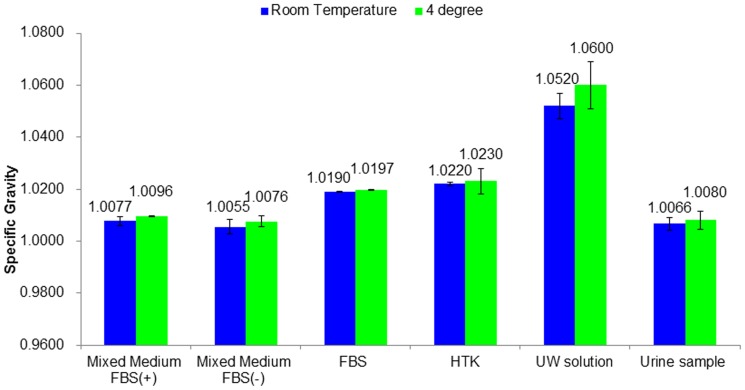
Gravity of different solution and urine samples. The gravity of nine urine samples from three different donors and five different preservation solutions were determined at room temperature and 24 hours storage in 4 degree. The specific gravity of UW solutions was the highest as 1.052 in room temperature and increased to 1.060 after 24 hours storage in 4 degree which was higher than the urine samples (1.0066 and 1.0080 in room temperature and after stored 24 hours in 4 degree respectively), and the USC culture medium without FBS's specific gravity was the lowest one (1.0055 and 1.0076 in room temperature and after stored 24 hours in 4 degree respectively).

To further study the mechanisms of preservation, the specific gravity of urine samples and preservation solutions were measured at room temperature and after being stored 24 hours at 4°C. The specific gravity of UW solutions was the highest; it was 1.052 at room temperature and increased to 1.060 after 24-hours storage in 4°C, higher than the urine samples (1.0066 and 1.0080 in room temperature and after being stored for 24 hours at 4°C, respectively). The USC culture medium's specific gravity was the lowest (1.0055 and 1.0076 at room temperature and after being stored 24 hours at 4°C, respectively). The higher gravity of UW solutions might be a reason why no cells survived because of cell membrane injuries (Fig. 1).

### Cell morphology, growth patterns, and cell surface marker expression

**Figure 2:**
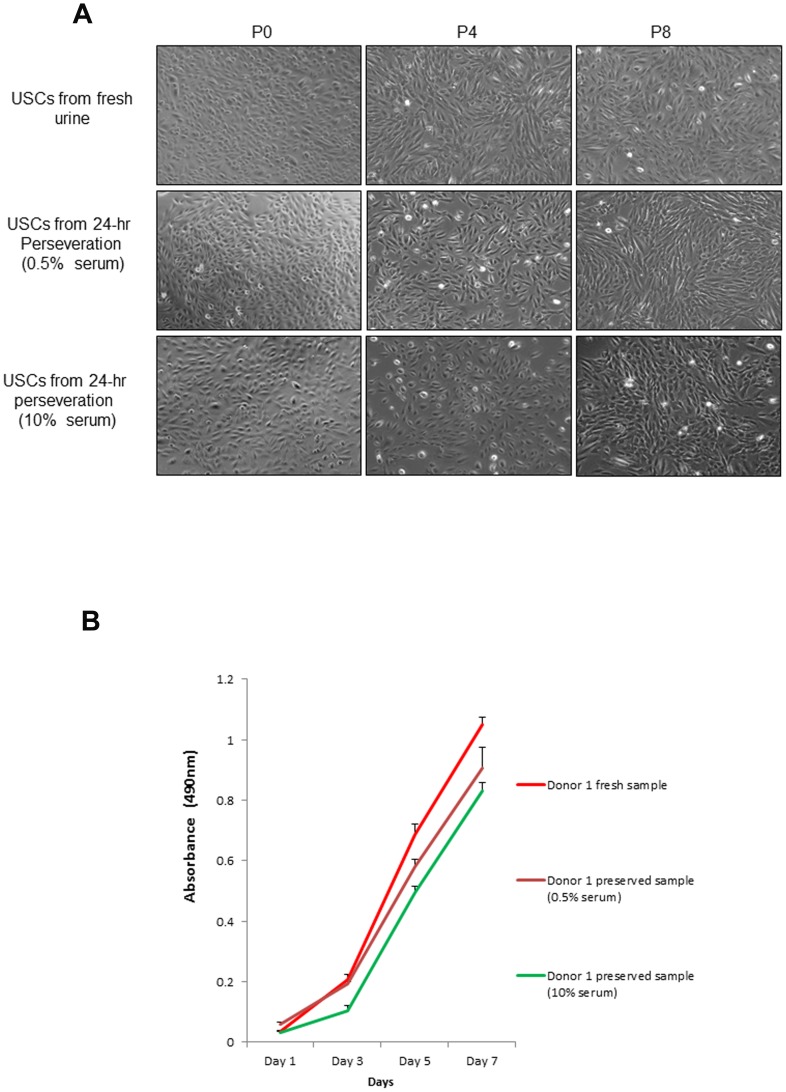
The cell morphology and cell growth curve of USCs from fresh urine and 24 hr preservation. (**A**) Cell morphology of the USCs preserved in culture media in different percentage of serum was similar to that of fresh USCs. (**B**) Cell proliferation curve showed cell growth pattern of 24-hr preserved USCs that was similar to that of fresh USCs. Images at 100×.

**Figure 3:**
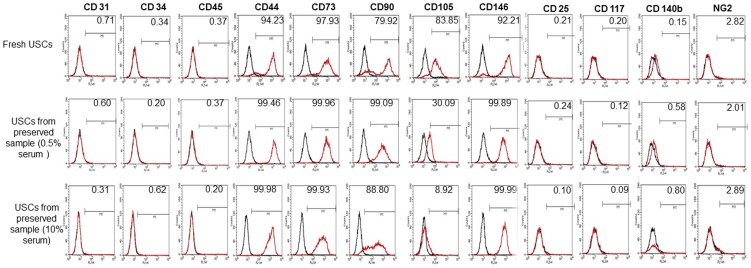
Cell surface marker expression of fresh USCs and 24-hr preserved USCs assessed by flow cytometry. Both fresh USCs and 24-hr preserved USC cells at passage 2 were strongly positive for the mesenchymal stem cell markers, such as CD44, CD73, CD90, CD 146 and CD105, and negative for the hematopoietic stem cell markers such as CD31, CD34 and CD45.

The cell morphology and growth pattern of the 24- hours preserved USC clones were similar to those obtained from fresh USCs (Fig. 2A, B). The cell clones all displayed “rice grain”-like morphology in fresh and 24-hours preserved urine samples. They had a bright, uniform and compact appearance, although USC clones appeared 1–2 days later in primary culture from 24-hours preserved samples than the cells from fresh urine. USCs from fresh and 24-hours preserved samples all could extensively expand *in vitro*. The average PD from passage 0 forward to passage 8 of fresh USCs was 49.5±7.2. On the other hand, 24-hours preserved USCs displayed an average PD of 47.5±5.8 with 0.5% serum and 46.9±6.7 with 10% serum to passage 8, respectively. The average DT was 32.2±13.51 hrs in 24-hours preserved samples with 10% serum, 28.3±8.55 hrs in 24-hours preserved samples with 0.5% serum, and 25.6±6.32 hrs in fresh sample (Table 3). There was no significant difference between these three groups. When immunophenotyping for surface antigens by flow cytometry was performed, fresh and 24-hours preserved clonal lines showed similar results. Both fresh and 24-hours preserved USCs were strongly positive for the mesenchymal stem cell markers CD44, CD73, CD90; weakly positive for CD105; and negative for the hematopoietic stem cell markers CD31, CD34, and CD45. Furthermore, these cells also expressed pericyte membrane marker CD 146 (Fig. 3).

**Table 3:** Comparison of growth characteristics of urine-derived cells from fresh urine and 24-hours storage urine sample.

	*Population Doubling with Passage (p)/Doubling Time (DT) in hours*									PD/Average DT (hour)
	*P1/DT*	*P2/DT*	*P3/DT*	*P4/DT*	*P5/DT*	*P6/DT*	*P7/DT*	*P8/DT*	*P9/DT*	
Fresh Urine	12.2/23.4	5.7/25.1	4.2/23.6	4.7/19.8	4.8/19.3	4.4/21.6	4.4/26.9	4.5/31.5	4.2/38.9	49.5±7.24/25.6±6.32
24 hours preserved Sample(0.5% serum)	12.8/27.9	5.0/28.6	4.4/15.8	3.5/19.7	4.6/21.7	4.7/30.5	4.7/29.1	3.5/41.1	4.0/40.3	47.5±5.86/28.3±8.55
24 hours preserved Sample(10% serum)	12.2/23.5	5.1/28.1	4.7/15.8	4.7/19.9	4.1/23.6	4.5/31.1	3.2/50.3	4.0/46.9	4.1/50.3	46.9±6.75/32.2±13.51

### Bipotent differentiation of preserved USCs

**Figure 4:**
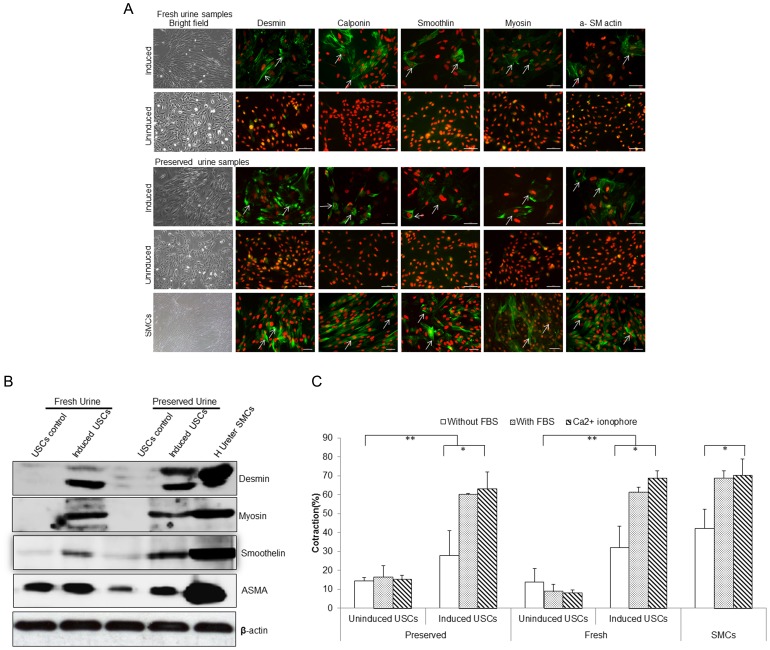
Smooth muscle differentiation of USCs. (**A**) Myogenically differentiated USCs from fresh urine and preserved urine expressed smooth muscle markers (such as desmin, myosin smoothelin, calponin and α-smooth muscle actin) that were similar to the ureter SMCs, while few cells displayed the same specific staining in un-induced USCs assessed by immunofluorescent staining. Scale bar = 50 uM. SMCs = ureter smooth muscle cells; USCs = urine derived stem cells. (**B**) Preserved USCs and fresh USCs 14 days after myogenic differentiation expressed about one-half to two thirds the amount of smooth muscle-specific protein (i.e. smoothelin, desmin and myosin ) compared to control (ureter SMCs) assessed by Western blot analysis. (**C**) Preserved USCs and fresh USCs displayed a strong contraction to calcium-ionophore A21387 (10^−5^ M) (61.4% and 60.2%, respectively) similar to the response of ureter SMC (68.9%). Non-induced USCs from fresh or preserved urine specimens lacked responses to calcium-ionophore in contractility analysis.

**Figure 5:**
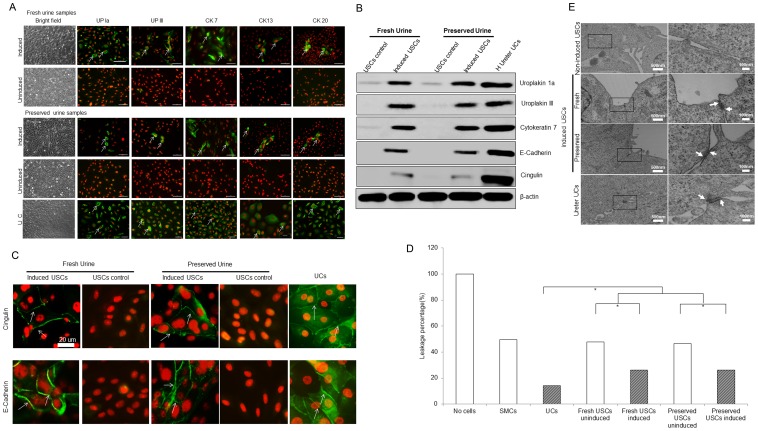
Urothelial differentiation of USCs. (**A**) Preserved USCs and fresh USCs 14 days after urothelial differentiation expressed urothelial cell markers (uroplakin Ia/III, CK7, CK13 and CK20) that were similar to the urothelial cells, while few uninduced USCs expressed these markers assessed by immunofluorescent staining. Scale bar = 50 uM. (**B**) Preserved USCs and fresh USCs expressed almost same amount of urothelial-specific proteins (uroplakin Ia/III, CK7, E-cadherin and cingulin) assessed with Western blotting. (**C**) Preserved USCs and fresh USCs displayed tight junction markers (cingulin and E-cadherin) on cell membrane boundaries (arrow) between cells detected by immunofluorescent staining. Scale bar = 20 uM. (**D**) Preserved USCs and fresh USCs exhibited tight junction-desmosomes (arrows) between two adjacent cells, whereas non-induced fresh USCs and preserved USCs showed desmosomes when examined by transmission electron microscopy (TEM). (**E**) A barrier function assay performed by using a fluorescent tracer on confluent cells cultured on inserts. Both preserved USCs and fresh USCs showed similar leakage protection that was significantly better than uninduced USCs (*p*<0.05).

To test whether 24-hr preserved USCs displayed at least bipotential differentiation capability, single clones of fresh and preserved USCs (p2) were subjected to myogenic as well as uroepithelial differentiation conditions for 14 days. A distinct morphologic change was evident by day 14 in each culture in both types of cells. The long, spindle-shaped morphology of stromal cells was evident in the fresh and 24-hours preserved USCs in myogenic differentiation medium, and a cuboidal phenotype all appeared in fresh and 24-hours preserved USCs in the uroepithelial differentiation medium. The bipotential differentiation capacity of fresh and 24-hours preservation USCs was also confirmed by the expression of specific proteins visualized by immunofluorescent staining for desmin, myosin, ASMA, calponin, and smoothelin for myogenic differentiation (Fig. 4A) and uroplakin-Ia (Up-Ia), Up-III, CK7, and CK13 for urothelial differentiation (Fig. 5A) and by analysis of the expression of lineage-specific proteins with Western blotting (Fig. 4B and 5B).

We used cell-collagen contractility analysis to measure the cell functionality of smooth muscle differentiated USCs (Fig. 4C). After mechanically releasing the cell-collagen gel lattices, contractile action was assayed by measuring the diameter before and after release. The gels mixed with smooth muscle differentiated from fresh and preserved USCs and ureter SMCs showed an average baseline contraction of 61.4%, 60.2%, and 68.9% in DMEM with 10% fetal bovine serum (FBS) separately, whereas gels from non-induced fresh and preserved USCs only showed about 8.9% and 16.1% contraction to serum. On the other hand, smooth muscle differentiated USCs showed a strong contraction to calcium-ionophore A21387 (10^−5^ M) [13] similar to the response of ureter SMC (Fig. 4C). Non-induced USCs lacked responses to calcium-ionophore. Calcium-ionophore A21387 is considered to be a unique smooth muscle agonist, as it causes a rapid contraction of smooth muscle cells but not fibroblasts [13].

To assess barrier function of UC-differentiated USCs, specificity of tight junction marker expression (E-cadherin and cingulin) was confirmed by Western blotting (Fig. 5B) and immunofluorescence staining (Fig. 5C). All tight junction markers stained the cell membrane boundaries between cells, and staining was even more pronounced upon induction (Fig. 5C). Using a fluorescent tracer on cells cultured on inserts revealed at least 50% decrease in leakage or protection after 3 h in vitro (Fig. 5D). To further determine the tight junction of urothelial differentiated USC clones from fresh urine samples and 24-hr mixed USC media preserved samples, TEM analysis was performed. The desmosomes were observed in both UC and induced USC clones and did not show significant differences, whereas non-induced samples fresh and preserved USCs did not show desmosomes (Fig. 5E).

### Telomerase activity of preserved USC

**Figure 6:**
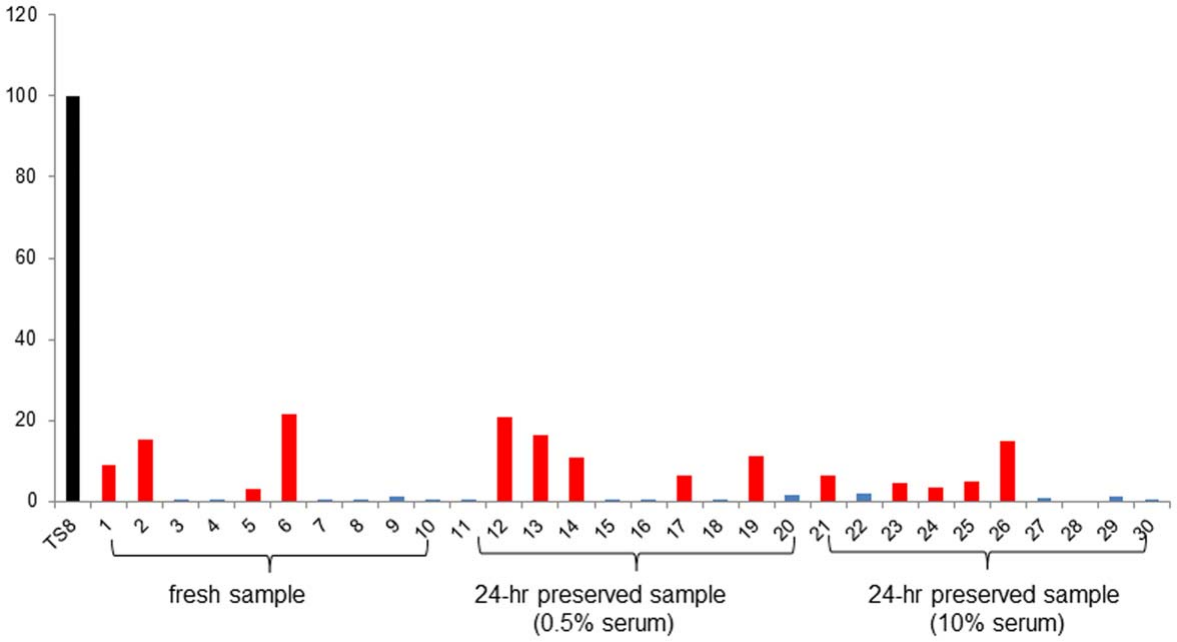
Telomerase activity in fresh USCs and 24-hr preserved USCs. Four of 10 fresh USCs, 5 of 10 preserved USCs (both preserved media with 0.5% and 10% FBS), respectively, showed significant telomerase activity (red bars). TS 8 were the positive control.

Relative telomerase activity was measured in 30 single USC clones from fresh and 24-hours preserved urine samples from one individual at passage 2, compared to TS8 as positive control. Four of 10 clones in fresh samples (40%), five of 10 clones in 24-hours preserved samples with 0.5% serum (50%), and five of 10 clones in 24-hours preserved samples with 10% serum (50%) showed significant higher telomerase activity, defined as more than twice the background value (Fig. 6). There were no significant differences between fresh and 24-hours preserved USCs in relative telomerase activity assays.

### Karyotyping of preserved USCs

Karyotype analysis was performed to test the chromosomal stability of USC clones from fresh urine samples and 24-hours preserved samples after serial cultures. Both USC lines all displayed a karyotype of 1 X and 1 Y chromosome, as expected for a male donor, and a normal diploid (2n = 46) complement of autosomes. No multiploidy or obvious chromosomal rearrangements at metaphase were detected by Giemsa bandings at passage 4 of both USC clones.

## Discussion

Cell turnover in physiological conditions indicates that differentiated or aged cells are naturally reduced by apoptosis and replaced by the division progeny of adult stem cells. The rate of cell turnover depends on each type of tissue or organ [14]. For example, intestinal epithelium is restored every 3–5 days in mammals [15], whereas stratum corneum replacement takes about 14 days and 28 days for replacement of the entire epidermis [16]. Urothelial cells display a much slower turnover rate of many weeks, and are basically quiescent until injury. Our hypothesis is that the more urine derived cells, including urothelial cells, renal tubule epithelial cells and others can be shed off from the urinary tract system into urine in the taller individuals due to the body size. We found a turnover rate of approximately 6×10^4^ cells in the urinary tract system over 24 hours, depending on the individual's age and height. Cells from older and taller donors shed off more cells into their urine samples. Although a large of experiment samples were not performed in the present study, the preliminary data showed a strong correlation between numbers of shed cells and individual height and age. Among these total numbers of cells, the living cells were about 4×10^3^ cells (13.7% of the total cells in urine). We used urine from healthy men, since voided urine from women is more likely to be contaminated with epithelial cells or vaginal bacteria. In addition, more cells may be present in urine collected by urethral catheter due to catheter induced injury of the bladder and urethral mucosa. Therefore, it is easier to collect the urine sample and derive more representative cells when cells are collected from voided urine than via a urethral catheter.

There are various sources of autologous mesenchymal stem cells [17], [18], [19], such as bone marrow and adipose tissue. However, our purpose was to identify a cell source with high self-renewal and multi-potent differentiation capacities that can be obtained via a simple and non-invasive approach. We recently found that a subpopulation of cells isolated from voided urine or urine from upper urinary tract [5] possess stem cells features, i.e. are highly expandable and have multi-lineage differentiation capability [3], [4], [5], [7], [9]. These urine-derived stem cells are capable of multipotent differentiation to mesoderm lineages (i.e. SMCs [4], [5], [7], [9] and endothelial cells [6]) and endoderm lineages such as urothelial cells [4], [5], [7], [9].

Our previous studies showed that USCs can survive for only a few hours in urine without any preservation, and eventually all the cells died within 24 hours after the urine was discharged from the body. Urine itself is unfavorable for cell survival due the lack of nutrients, metabolic waste material that might be toxic, osmotic pressure, and a non-physiological pH value. This toxic environment impairs cellular membrane and causes cellular lysis. Two strategies can be used to preserve cells and improve the results of their necessary storage. One is cryopreservation, to spin down the cells from urine and freeze them in dimethyl sulfoxide (DMSO) in liquid nitrogen immediately, which requires the appropriate facilities to isolate and store cells. The other strategy is to add the preservation medium in urine and store whole urine samples at 4°C to retain cell membrane stability, slow down metabolic processes, and prevent cell lysis. The advantage of this approach is that no special equipment or processes are needed to isolate and freeze cells, which could be more convenient for patients.

An effective and simple preservation method is greatly advantageous for accumulating more USCs for necessary storage or for USC distribution within a restricted time period for potential clinical application. When large amounts of USCs at early stages are needed rapidly for cell-based therapy, collecting more urine samples to preserve living USCs is necessary. MSC at earlier passages possess greater plasticity and higher growth potential [20]. It would be ideal for cell therapy to obtain USCs at as early passage as possible, since cells often lose differentiation function and proliferation capacity with number of passage in cultures. Our previous studies demonstrated that 5–10 USC colonies can be formed per fresh 100 ml urine. Each single USC clone can generate four million cells at passage 3 within 2 weeks [5], and the USCs at early passage can be more efficiently differentiated into urothelial and smooth muscle cells [4], [5], [7], [9].

It would be beneficial and convenient for patients to collect their urine samples, refrigerate them at home, and then bring the samples to the hospital rather than keeping them as inpatients. In addition, using urine samples stored for 24 hours allows batching of processing, which minimizes costs and decreases the possibility of contamination in each step of the cell isolation process. The ability to preserve cells in urine permits transportation of the cells if necessary for cell isolation, characterization and culture of USCs for research or future clinical use.

In this study, we optimized preservation techniques to maintain the maximum amount of cell viability and optimal stem cell properties, i.e. self-renewal and multi-potency during cell preservation in urine. In each 100 ml of urine samples, 3–4 USC clones existed in 24-hours preserved urine, 4–5 clones in 12-hours preserved urine, and about 6–7 USC clones in fresh urine. Overall, in about 100 USC clones, nearly 50–80% of fresh USCs can be preserved from adult urine during 24-hours storage by these preservation methods. USCs can be obtained from each donor. Although USCs cannot be obtained from each urine sample, they can be obtained from about 70% of preserved urine specimens of each individual. Importantly, the quality of stored USCs was the same as fresh USCs in each area tested, consistent with our previous study [6], [21]. Like fresh USCs, the preserved USC clones initially grew a single rice-grain-like cell and then formed a cluster of 6–9 cells in the initial primary culture. They underwent more than 47 population doublings compared to fresh USCs, with nearly 50 population doublings. The preserved cells expressed mesenchymal stem cell [22], [23] and perictye markers [24], such as CD44, CD 73, CD90, and CD105, CD146, but not hematopoietic stem cell markers [25], [26] such as CD31, CD34, and CD45. Half (5 of 10) preserved cell clones had high telomerase activity and 4 of 10 fresh USCs had telomerase activity; all had normal karyotypes. Prominently, the preserved USCs maintained bi-potent differentiation capacity. After storage, differentiated USCs expressed myogenic-specific genes and proteins [27], [28] such as desmin and myosin contractile function when exposed to myogenic differentiation medium. They also expressed urothelial genes and proteins [29], [30] such as uroplakin I and III a, and tight junction genes, proteins such as E-cadherin and cingulin when exposed to urothelial differentiation medium. Furthermore, the urothelially differentiated cells possessed tight junction ultra-microstructure and barrier function.

To optimize preservation conditions, six different solutions were tested, and USC culture medium with serum was the best environment for USCs during simple cold storage, compared to serum alone, culture medium alone or organ preservation solutions. The numbers of the cells declined markedly using other solutions. As the densities of cells and preserved medium (>1) are both slightly higher than urine, the cells sank on the bottle of containers with medium at 4°C during storage due to gravity. Most cells were immersed in the culture medium. This might be why a small amount of preserved medium (about 10–15% of urine volume) can retain the USC clones.

Maintaining stability of the cellular membrane is critical in preservation for stem cells in urine, given its limited content of nutrients and oxygen. Although it is unknown how culture medium with serum provides cell protection, multiple factors might be involved in stabilization of the cell membrane. For example, the culture medium may offer a favorable osmotic concentration, or the presence of serum and supplements in the medium (e.g. hydrocortisone and insulin) may help to maintain cell membrane stability, prevent edema and chemical toxicity [31]. Therefore, the combination of culture medium and serum provided better conditions to preserve USC clones. In addition, the lack of glucose in the medium may prevent cellular metabolism. Furthermore, hypothermic storage at 4°C reduces the rate of cell metabolism and oxygen consumption, and thus reduces cellular impairment.

Compared to organ solutions, culture medium provided better conditions for cell preservation in these experiments. Although both HTK Solution [32], [33] and University of Wisconsin solution [33], [34] have been successfully used in preservation of donor kidneys, livers, pancreas, and hearts, in our studies, neither solution retained USCs better than the USC culture medium. In addition, both media are very expensive and might not be recommended to use in preservation of cells in body fluid.

In summary, human USCs are a potential cell source for cell therapy in the urinary tract system. It would be beneficial to preserve the USCs or cells in urine and also retain cell quality and function of USCs during necessary storage and transportation. In this study, we demonstrated that USCs can be obtained from each individual's preserved urine specimens. The cell quality and function of preserved USCs are the nearly same as the fresh USCs in cell morphology, cell growth patterns, expression of stem cell surface makers, self-renewal capacity, differentiation capacity, expression of telomerase activity, and chromosome stability. Culture media with a minimum of serum added into the urine significantly increased cell viability and maintained membrane integrity of USCs compared with cells in organ preservation solutions. This preservation approach is simple, effective and low cost, which makes it possible to transport living USCs in the urine, or possibly or to preserve cells contained in other body fluids for transportation or for short-term storage.

## Supporting Information

Table S1 Details of antibodies used in this study.
(DOC)

Table S2 The average number of cells in 24 hour storage of urine samples on a series of 3 days. The average total number of cells derived from 166 urine samples from 12 healthy adult donors was 58,560±27,980 cells (range 25,226 to 107,543 cells) in 1,680±846 ml urine in a 24-hr period. The average living cells in that period was 8,076±4,784 (range 3,070 to 15,820 cells), as measured by trypan blue exclusion. The ratio of live cells to total cells was 13.65±4.96% (range 5.96%∼22.53%).
(DOC)
