# Supplementary material for: Self-Renewal and Differentiation Capacity of Urine-Derived Stem Cells after Urine Preservation for 24 Hours
Source: PLoS One. 2013 Jan 18;8(1):e53980. doi: 10.1371/journal.pone.0053980 (PMC3548815; doi:10.1371/journal.pone.0053980)
Supplement: Table S1 — Details of antibodies used in this study. (DOC) [file pone.0053980.s001.doc]

| Table S1. Details of antibodies used | | | | | | |
| --- | --- | --- | --- | --- | --- | --- |
| Primary Antibody | Source/Catalog # | Antibody Dilution | | | | |
|  |  | IF | WB | | FACS | |
| Smooth muscle markers:  Desmin | Abcam/ab15200 | 1:200 | | 1:500 | | - |
| Myosin (smooth) | Sigma/M7786 | 1:50 | | 1:500 | | - |
| α-smooth muscle actin | Abcam/ab5964 | 1:200 | | 1:1000 | | - |
| Calponin (G-10) | Santa Cruz/sc136987 | 1:50 | | - | | - |
| Smoothelin | Abcam/ab8969 | 1:100 | | 1:250 | | - |
| Urothelial cell markers:  Uroplakin-Ia (C-18) | Santa Cruz/sc15173 | 1:50 | | 1:200 | | - |
| Uroplakin-III (M-17) | Santa Cruz/sc15186 | 1:50 | | 1:200 | | - |
| Cytokeratin-7 (N-20) | Santa Cruz/sc17116 | 1:50 | | 1:200 | | - |
| Cytokeratin-13 (Ks13.1) | Santa Cruz/sc101460 | 1:25 | | - | | - |
| Cingulin (C-17) | Santa Cruz/sc46572 | 1:100 | | 1:200 | | - |
| E-cadherin (G10) | Santa Cruz/sc8426 | 1:50 | | 1:200 | | - |
| β-actin(C4) | Santa Cruz/sc47778 | - | | 1:2000 | | - |
| Cell surface markers:  CD31-FITC | BD Pharmingen/560984 | - | | - | | 1:5 |
| CD34-FITC | BD Pharmingen/560942 | - | | - | | 1:5 |
| CD44-FITC | BD Pharmingen/560977 | - | | - | | 1:5 |
| CD45-FITC | BD Pharmingen/560976 | - | | - | | 1:5 |
| CD90-FITC | BD Pharmingen/555595 | - | | - | | 1:5 |
| CD73-PE | BD Pharmingen/561014 | - | | - | | 1:5 |
| CD146-PE | BD Pharmingen/561013 | - | | - | | 1:5 |
| CD105-PerCP-Cy5.5 | BD Pharmingen/560819 | - | | - | | 1:20 |
