# Supplementary material for: Self-Renewal and Differentiation Capacity of Urine-Derived Stem Cells after Urine Preservation for 24 Hours
Source: PLoS One. 2013 Jan 18;8(1):e53980. doi: 10.1371/journal.pone.0053980 (PMC3548815; doi:10.1371/journal.pone.0053980)
Supplement: Table S2 — The average number of cells in 24 hour storage of urine samples on a series of 3 days. The average total number of cells derived from 166 urine samples from 12 healthy adult donors was 58,560±27,980 cells (range 25,226 to 107,543 cells) in 1,680±846 ml urine in a 24-hr period. The average living cells in that period was 8,076±4,784 (range 3,070 to 15,820 cells), as measured by trypan blue exclusion. The ratio of live cells to total cells was 13.65±4.96% (range 5.96%∼22.53%). (DOC) [file pone.0053980.s002.doc]

| Table S2. The average number of cells in 24 hr storage of urine samples on a series of 3 days | | | | | | | |
| --- | --- | --- | --- | --- | --- | --- | --- |
| Donor # | Average cell Number/24hr (3 days) | Ratio of living cells to total cells (%) | Urine volume (ml)/24hr | Age(yrs) | Weight (kg) | Height (meter) | BMI (Body Mass Index) |
| 1 | 76290±19218 | 20.31±2.57 | 2,180±242 | 45 | 75 | 1.72 | 25.35 |
| 2 | 86804±28956 | 16.09±3.77 | 2,306±23 | 54 | 87 | 1.80 | 26.85 |
| 3 | 85999±9670 | 13.52±2.63 | 1,856±436 | 45 | 75 | 1.75 | 24.48 |
| 4 | 35900±1457 | 22.53±1.74 | 2,065±176 | 42 | 70 | 1.72 | 23.66 |
| 5 | 107543±33790 | 12.23±4.02 | 2,290±242 | 30 | 59 | 1.71 | 20.17 |
| 6 | 30010±12656 | 14.25±2.15 | 1,311±266 | 36 | 82 | 1.73 | 27.39 |
| 7 | 68404±33809 | 5.96±1.22 | 1,481±400 | 42 | 83 | 1.64 | 30.85 |
| 8 | 25226±15138 | 18.76±4.03 | 1,251±304 | 20 | 61 | 1.82 | 18.41 |
| 9 | 48270±19860 | 10.42±0.43 | 1,496±353 | 28 | 63 | 1.74 | 20.80 |
| 10 | 29204±6513 | 10.97±2.87 | 582±32 | 35 | 54 | 1.68 | 19.13 |
| 11 | 50579±18577 | 8.88±2.60 | 1,308±106 | 36 | 73 | 1.70 | 25.25 |
| 12 | 46822±7810 | 9.88±2.62 | 2,040±144 | 27 | 65 | 1.68 | 23.03 |
| Total Average | 58,560±27980 | 13.65±4.96 | 1,680±846 | 36.6±9.5 | 70.5±10.4 | 1.72±0.05 | 23.78±3.69 |
